# Influence of Sugarcane Bagasse Ash and Silica Fume on the Mechanical and Durability Properties of Concrete

**DOI:** 10.3390/ma15093018

**Published:** 2022-04-21

**Authors:** William Earl Farrant, Adewumi John Babafemi, John Temitope Kolawole, Biranchi Panda

**Affiliations:** 1 Department of Civil Engineering, Stellenbosch University, Stellenbosch 7602, South Africa; 20714009@sun.ac.za; 2 School of Architecture, Building and Civil Engineering, Loughborough University, Loughborough LE11 3TU, UK; J.T.Kolawole@lboro.ac.uk; 3 Department of Mechanical Engineering, Indian Institute of Technology Guwahati, Assam 781039, India

**Keywords:** sugarcane bagasse ash, silica fume, durability, compressive strength, oxygen permeability index

## Abstract

Cement production is environmentally unsustainable due to the high anthropogenic carbon emissions produced. Supplementary cementitious materials (SCMs), derived from the by-products of different industries, have been deemed an effective way to reduce carbon emissions. The reduction in carbon emissions is achieved by lowering the clinker factor of cement, through a partial replacement with an SCM. Sugarcane Bagasse Ash (SCBA) is produced as an agricultural waste from the sugarcane industry and has gained a lot of attention for being a feasible and readily available pozzolanic material, underutilised as an SCM. This study evaluates alkali-activated sugarcane bagasse ash’s mechanical and durability performance, at varied contents, in binary blended cement concrete and ternary blended cement concrete containing silica fume (SF). Potassium Hydroxide (KOH), used as the alkali activator, is intended to enhance the reactivity of the ash, with the possibility of a high-volume SCBA content. The mechanical performance was investigated by compressive and split tensile strength tests, and durability performance was investigated using the Oxygen Permeability Index (OPI) test. In addition, a micro-CT porosity test was conducted to assess how the microstructure and porosity of the concrete affect the mechanical and durability performance. The results indicated that using SCBA in a ternary blend with SF can significantly improve the overall performance and create less porous concrete. At 30% SCBA and 10% SF replacement, the performance tests revealed the highest mechanical strength and the lowest permeability, outperforming the control concrete and the binary blended cement concrete containing only SCBA.

## 1. Introduction

Global warming has become a very big concern to the future of civilisation and creating a sustainable and environmentally conscious method of development is paramount. Ordinary Portland Cement (OPC) is one of the major ingredients used in concrete. However, OPC production produces copious amounts of carbon dioxide (CO_2_) through clinker production [1]. This contributes to cement’s large environmental footprint. For example, 5% and 7% of the global anthropogenic CO_2_ and greenhouse gasses (GHG) are from the cement industry [2,3]. The environmental footprint emanates from both material and energy-associated processes [4]. Supplementary cementitious materials (SCMs) are beneficial for reducing cement’s carbon emissions. SCMs reduce the clinker-to-cement ratio by partially replacing the cement content. This indirectly reduces carbon emissions. Other benefits associated with the use of SCMs in the optimum proportion include an increase in concrete’s mechanical and durability performance [5]. SCMs are chosen based on their favourable chemical characteristics, containing sufficient amounts of aluminosilicate minerals [6], with potential for good pozzolanic activity by combining with the cement hydration product (Ca(OH)_2_) in a suitable alkaline environment. Sugarcane Bagasse Ash (SCBA) is produced as a secondary product from the sugarcane industry. Bagasse is produced during the extraction of sugar from sugarcane; this bagasse is considered a waste product that is then burnt as a fuel for electricity generation [7]. After the bagasse is burnt, the final by-product is SCBA. SCBA has good pozzolanic activity due to very high silica content, and this pozzolanic potential of SCBA can be maximised if processed correctly [8]. Globally, over 100 countries produce about 2.1 billion metric tons of sugarcane, and in South Africa, the annual production of sugarcane is about 15.07 million metric tons [3]. In regional areas, such as South Africa, the production of SCBA outweighs other biosilica sources, such as rice husk ash, with the production of 115 thousand metric tons.

The SCBA obtained from electric plants cannot be directly used as a SCM due to unburnt particles [5] and the presence of high crystalline phases [9]. Different processing methods, such as grinding and calcination, enhance SCBA’s pozzolanic potential [10]. Silica fume (SF) is a well-established SCM, produced during the silicon smelting process [11]. The addition of SF with a natural pozzolan as a cement replacement has been shown to boost the performance of concrete through microstructure enhancement and an improvement in the aggregate-matrix bond [12].

The use of SCBA in cementitious composites is limited to low volumes of 20 to 25% due to a substantial decrease in performance at higher content [5,13,14,15]. This decrease is supposedly due to the delayed pozzolanic reaction of SCBA at early ages [16]. Therefore, there is a scarcity of literature on concrete’s mechanical and durability performance using SCBA at 30% and above replacement. The motivation of this study lies in increasing the volume of SCBA in cementitious composites, without compromising the performance. Therefore, the study sets out to incorporate 30% and higher volume of Portland cement replacement to enhance the sustainability of ensuing cementitious composites.

Possible ways to counter these effects are by chemical activation, such as potassium hydroxide (alkali-activated SCBA) and blending with SF. Alkali activation can potentially improve the early age pozzolanic reactivity of SCBA by contributing to an early alkaline environment, compared to that later provided by cement hydration—Ca(OH)_2_ end products [17]. The alkali (such as KOH) helps dissolve the SCM’s aluminates and silicates and form cementitious hydrates [18,19]. It is roughly estimated that for every 1 mol of KOH produced, an indirect 1.5 mol of CO_2_ is generated for electrolysis [20]. Using this study as an example, only 1% of KOH relative to the binder is required for activation, presumably negligible in the carbon footprint of the ensuing composite.

The addition of SF (micro-silica) can provide synergetic effects in improving the microstructure and nucleation sites to form cement hydration. SF is known to be highly amorphous and finely grained; that is, improved pozzolanic activity and micro-filler effects for improved reactivity, pore size, aggregate interfacial refinement, and better packing density [8]. Combining both approaches can improve the mechanical and durability performance of SCBA blended cement concrete. However, this approach, allowing for a high volume of SCBA as SCM in cementitious composites, lags in the literature. The current study intends to add to the body of knowledge on the use of SCBA as SCM in this regard.

This study is poised to take advantage of the alkali activation and SF inclusion to assess the performance of concrete with a high volume of SCBA contents. This is done by replacing Portland cement (PC) of 30% and 50% with alkali-activated SCBA and blending with 10% SF as separate mixtures. This is to understand how these two SCMs interact with each other and the potential to use SCBA as an SCM at these relatively high replacement percentages. It should be noted that this study does not intend to isolate and quantify the effect of alkali activation; hence, the activator content was kept constant for all mixes, except the control. The performance of the mixtures was assessed by compressive and splitting tensile tests for mechanical strength and oxygen permeability index (OPI) tests for durability. To explain the obtained results for the performance of the mixtures and identify the influence of the SCMs, characterisations of the SCMs were undertaken, mortar samples of each mix were characterised, and micro-CT scans were carried out on the concrete samples to assess the microstructure.

This study is significant as it shows how using a SCBA replacement of 30% in a ternary blend with 10% SF can produce concrete that outperforms the binary blend at the same SCBA replacement and control (Portland cement), in terms of mechanical and durability performance. By using this alkali-activated ternary blend, the limitations of using SCBA replacements up to a maximum of 20–25% can be overcome. This research is fundamental in producing environmentally sustainable concrete for the future. It indicates that up to 40% of the cement can be replaced while producing no negative effect on performance. This will significantly reduce the clinker factor of the cement and, consequentially, reduce carbon emissions.

## 2. Materials and Methods

### 2.1. Materials 

The cementitious materials comprised PC (CEM II 52.5N), processed SCBA (90% passing through the 45 microns sieve size), and SF (Chryso densified silica fume with a specific surface area of 13,000–20,000 m^2^/kg). SCBA was sourced as raw ash from Kwa-Zulu Natal in South Africa. Graded river sand (Malmesbury sand) was used as the fine aggregate, and 19 mm quarry stone was used as the coarse aggregate. Potassium Hydroxide (KOH) was used as the alkali activator and intended to enhance the pozzolanic reactivity. KOH was chosen as it has an ionic size K^+^ of 152 pm. This is very small and will accelerate the dissolution process of the aluminosilicate minerals [21]. The other admixture used was a superplasticiser, CHRYSO^®^ Fluid Premia 310 (Issy-les-Moulineaux, France), a modified polycarboxylate polymer. This plasticiser was required to achieve the correct workability while maintaining a constant water-to-border ratio. The materials used and the characteristics of each are shown in Table 1, where RD and FM represent the relative density and fineness modulus, respectively.

### 2.2. Processing of Sugarcane Bagasse Ash (SCB)

The raw SCBA was obtained in wet form and dried completely using an industrial fan in the laboratory. During the collection of the ash for calcination, the sand content was mostly separated from the ash. The ash was calcinated at a temperature of 650 °C for 1 h and 30 min using a Gallenkamp Muffle Furnace (Cambridge, UK). Calcination has been shown to increase the pozzolanic activity of SCBA [8,10,22]. Kolawole et al. [8] showed that calcination at a temperature range between 600 °C and 700 °C for a 1-to-2-h duration has the potential to decrease the carbon content, decrease the loss on ignition percentage, as well as increase the pozzolanic oxides present in the material [23,24].

### 2.3. Concrete Mix Design

The mix designs were developed according to the Cement and Concrete Institute (C&CI) design method based on the ACI Standard 211.1-91 [25]. The water-to-binder (w/b) ratio of 0.5 was used for all mix designs, as this is a typical value used for conventional concrete. The cementitious material content was maintained at 400 kg/m^3^ throughout. The slump range of 50–90 mm was maintained using the superplasticiser. KOH was added to all mixes containing SCMs at a dosage of 1% with respect to the binder weight and then subtracted from the water mass to ensure that the w/b ratio was consistent. The mix proportions for a 1000-litre mix are shown in Table 2. The first mix (Mix 1) is the control, the second mix (Mix 2) contains a 30% SCBA replacement, the third mix (Mix 3) contains a 50% SCBA replacement, the fourth mix (Mix 4) contains 30% SCBA and 10% SF replacement, and finally, the fifth mix (Mix 5) contains 50% SCBA and 10% SF replacement. The slump was used as the workability test following SANS 5862-1:2006d [26] and kept within the category S2 (50–90 mm) of BS EN 206-1.

### 2.4. SCBA, SF, and Paste Characterisation

#### 2.4.1. XRF

X-ray fluorescence (XRF) analysis was used to obtain the chemical (oxide) composition of the SCMs (processed SCBA and SF) using a PANalytical AXIOS XRF spectrometer, Malvern Panalytical (Pty) Ltd., Aimelo, The Netherlands.

#### 2.4.2. XRD

Qualitative and quantitative X-ray diffraction (XRD) analysis was conducted on the raw SCBA, processed SCBA, and SF to determine the mineralogical characteristics of these cementitious materials. By this, the influence of SCBA calcination can be observed. In addition to these cementitious materials, the sample of each concrete mix was analysed at a curing age of 21 days. A backloading preparation method was used for the XRD analysis. Hence, the prerequisite for the testing procedure was that all samples were to be tested in powder form so that they could be placed into the backloaded sample holder. The (mortar) sample was prepared by hand grinding the concrete samples using a mortar and pestle and sifting out the coarse aggregate. The mortar samples were analysed to identify the hydration extent and observe the influence of the adopted approaches (alkali activation and SF blending).

The diffractograms were obtained using a Malvern Panalytical Aeries diffractometer, Malvern Panalytical (Pty) Ltd., Aimelo, The Netherlands. This diffractometer had a PIXcel detector, and fixed slits with Fe-filtered Co-Kα radiation. The phases were identified using X’Pert Highscore plus software, Malvern Panalytical (Pty) Ltd., Aimelo, The Netherlands, and the specific crystalline phase amounts (percentage of weight) were obtained using the Rietveld Method. The Internal Standard Method was used to obtain the amorphous phase amount (percentage of weight). Further, 20% addition of silicon was added as the internal standard as it is 100% crystalline and nonreactive with the cementitious materials and mortars. The addition of silicon as the internal standard allowed the phase quantity of the samples to be rescaled to 20% silicon. Thus, absolute phase quantities could be obtained, with the specific deviation to 100% weight percentage, constituting the resulting amorphous phase amount.

#### 2.4.3. SEM

Scanning electron microscopy (SEM) was carried out to determine the morphology of the SCMs (raw SCBA, processed SCBA, and SF). The equipment used to obtain the SEM images was a Zeiss MERLIN Field Emission SEM, Carl Zeiss AG, Jena, Germany, with a voltage of 10 kV.

### 2.5. Preparation of Test Specimens

#### 2.5.1. Specimen Preparation for Mechanical Strength Tests

SANS 5861-2:2006a [27] and SANS 5861-3:2006c [28] were followed to ensure the correct procedure for sampling, curing, and preparing test specimens for strength tests from the fresh concrete mixes. The compression and splitting tensile strength test specimens were cast in 100 mm cube moulds according to SANS 5860:2006a [29]. Compaction of the concrete in the moulds was carried out on a vibrating table. The concrete specimens were then placed in a temperature-controlled room for one day before being de-moulded and cured in water tanks at a temperature of 23 ± 1 °C. Both the compressive and split tensile strength tests were conducted at 7 and 28 days of curing.

#### 2.5.2. Specimen Preparation for the OPI Test

The preparation of the test specimens for the OPI test was done following SANS 3001-CO3-1:2015 [30], which required that the mould be used for durability index testing by a cube, with dimensions of 100 mm. The cubes used were then cored and cut to the specific dimensions being 30 mm thick and 70 mm in diameter. Furthermore, 28-day cured specimens had to be further dried at 50 °C for 7 days in an oven before testing.

### 2.6. Performance Test Methods

#### 2.6.1. Compressive Strength Test

The compressive strength test was conducted in accordance with SANS 5863:2006e [31]. This test involves using three cubes, all from the same sample of concrete. The test was performed on saturated concrete specimens; hence, testing commenced immediately following their removal from the water curing tanks. Before the cube was placed into the testing machine, it was cleaned, and all surface water was removed. The load was applied to the specimen at a constant rate of 0.3 MPa/s until failure occurred. The maximum load at failure was recorded, and the mean value of the three specimens was used per concrete sample.

#### 2.6.2. Split Tensile Strength Test

The split tensile strength test was conducted following SANS 6253:2006f [32]. This test involves using three cubes, all from the same sample of concrete. This test also requires that the specimens be tested in a saturated state and hence they were tested immediately following their removal from the water curing tanks. The specimen, as well as the test machine, was cleaned before conducting the test. When the specimen was placed into the machine, packing strips (plywood strips as per SANS 6253:2006f [32] dimensions) were carefully placed along the top and bottom of the specimens’ centres in the loading plane, which was on the opposite as-cast face. Loading was applied to the specimen at a constant rate of 0.03 MPa/s until failure occurred. The maximum load at failure was recorded, and the mean value of the three specimens was used per concrete sample.

#### 2.6.3. Strength Activity Index (SAI) Test

This test method was conducted in accordance with ASTM C311:2018 [33] and ASTM C618:2018 [34]. It was conducted to assess the pozzolanic activity of the SCBA and is an indirect testing method to assess pozzolanic activity. This test was conducted at curing ages of 7 days and 28 days. ASTM C311:2018 [34] specifies the amount of SCM replacement as 20% of the cement content by weight. As such, 30% was used in this study, as a mix design with only 20% SCBA replacement was not conducted. The SCM blended cement concrete must have 75% of the compressive strength of the control at the respective curing ages. The SCM must have adequate pozzolanic activity and be accepted as an SCM under the specifications of ASTM C618:2018 [34].

#### 2.6.4. Oxygen Permeability Index (OPI) Test

The OPI test was conducted per SANS 3001-CO3-2:2015 [35]. This test was achieved by measuring the pressure drop of pressurised oxygen passed through the concrete specimens inside a permeability cell. Each concrete specimen was situated in an airtight seal surrounded by a compressible collar placed into the permeability cell. After ensuring that only oxygen was inside the permeability cell and no leaks, the pressure was set to 10 ± 5 kPa. The pressure drop was measured using an automated pressure-recording device that recorded the data at 2-min intervals for enhanced accuracy over 6 h. Measurement was not stopped before 6 h, as no specimen reached 50 kPa before the allocated time. 

#### 2.6.5. Micro-CT Porosity Test

Porosity analysis is the method of determining the pore spaces or voids present inside the concrete. It is used to assess the concrete’s durability (and mechanical) properties for the transport properties. The Micro-CT (Micro Computer Tomography) scanner was a General Electric V TomeX L240 system, General Electric, Wunstorf, Germany. This system used an X-ray source and detector to obtain 2D images, which were then combined to create a 3D representation of the reconstructed 2D images [36]. The X-ray was set at 220 kV and 200 μA, and the voxel size set for analysis was 80 μm. The software used for analysis and 3D visualisation was Volume Graphics VGStudioMax3.4, Volume Graphics GmbH, Heidelberg, Germany.

This analysis was conducted to assess the porosity of the different concrete mixes. It could be analysed through qualitative visualisation of the spatial distribution of voids present in the concrete [37] and/or quantitative analysis. Detailed data processing and analyses can be found elsewhere [36]. The voids were characterised based on the information extracted from the individual pore spaces that made up each specimen.

## 3. Test Results and Discussion

### 3.1. SCBA, SF and Mortar Characterisation

#### 3.1.1. XRF

The results of the XRF analysis are shown in Table 3. Following ASTM C618:2018 [34], for SCBA to be classified as a Class N pozzolan (natural pozzolan), it must meet the requirements that stipulate an LOI of less than 10%, as well as the pozzolanic oxide composition being greater than 70% of the total oxide composition of the material. From the XRF results, SCBA’s pozzolanic oxides add up to 82.24%, and its LOI is 7.94%. Therefore, it meets the requirements that classify it as a suitable SCM. In addition, there is a high silica content present in both SCBA and SF, which should be of advantage to the pozzolanic potential of these materials. High LOI percentages can be detrimental to SCBA’s performance as an SCM, especially for curing ages of less than 90 days [38]. The LOI of the SCBA in this study is low, and this was achieved through the processing procedure conducted on the SCBA.

#### 3.1.2. XRD

**Figure 1:**
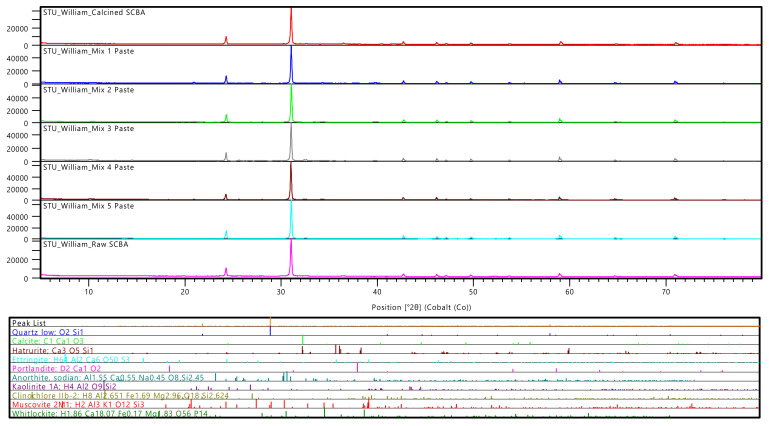
Diffractograms for raw SCBA, processed SCBA, and mortar.

**Figure 2:**
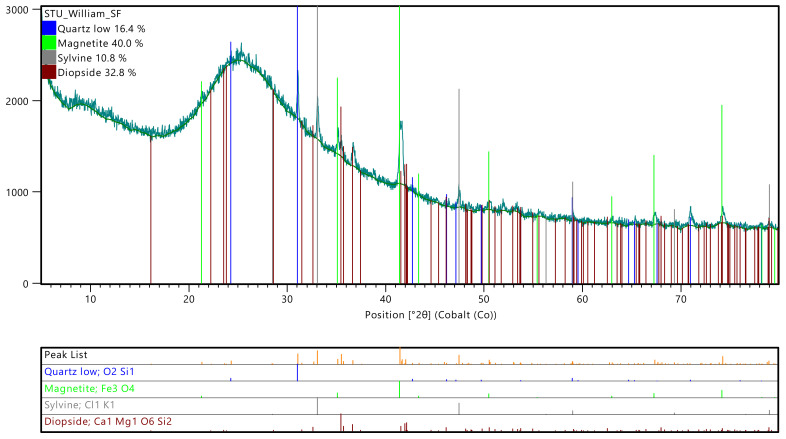
Diffractogram for SF.

The results from the XRD analysis are represented by mineral names, which correspond to the specific group of minerals, as shown in Table 4. In addition, the amorphous phases are represented by a weight percentage that is separate from the crystalline phases. XRD diffractograms are shown in Figure 1 and Figure 2.

From Table 4, processing the SCBA increased the amorphous weight percentage by about 13%. Crystalline silica phase (quartz) has also been reduced in the processed SCBA by about 11%. Effective pozzolanic reactions are primarily due to the amorphous or partially crystalline silica present and are adversely dependent on the crystallinity of the silica. In a study conducted by Corderio et al. [24], it was observed that decreasing the quartz content with an increase in amorphous content directly contributed towards higher reactivity. Therefore, the decrease in quartz and high amorphous content observed for the processed SCBA in this study are expected to contribute to the higher pozzolanic activity. The higher quartz content in the raw SCBA could also be due to sand contamination left on the sugarcane during harvesting [9]. The amorphous weight percentage of SF is 96.1%. With such a high amorphous content, it is expected to have high pozzolanic reactivity in concrete.

Portlandite is the natural form of calcium hydroxide (Ca(OH)₂) and is used up for pozzolanic reactions of SCMs. A pozzolanic reaction is from the amorphous and partially crystalline siliceous phases in the SCMs reacting with the calcium hydroxide produced during hydration to yield calcium silicate hydrate (C-S-H) gel [6]. From the table, the portlandite phase decreased with the increase in SCMs. The binary blends containing only SCBA replacement (Mixes 2 and 3) showed a decrease in portlandite, from 3.4% for control to 2% for Mix 2 and 0.8% for Mix 3. This indicates that the SCBA has pozzolanic activity by decreasing the calcium hydroxide phase. This pozzolanic activity of SCBA is later confirmed with the SAI results in Section 3.3.

A further decrease in the calcium hydroxide phase is observed in the ternary blends containing SCBA and SF (Mixes 4 and 5). Mixes 4 and 5 have a portlandite weight percentage of 0.4% and 0.2%, respectively. This proves a very high amount of pozzolanic activity in these mixes, as Mix 5 is close to 0% portlandite. Portlandite was decreased significantly more in the ternary blends than the binary blends at the same SCBA replacement percentage. This supports the fact that the amorphous nature of SF has significantly increased the pozzolanic activity during hydration. The combination of these two SCMs produced high reactivity. The high reactivity could also be attributed to the use of KOH as the alkali activator, as KOH helps raise the pH to a level that can start and maintain the pozzolanic reactions [6]. It should be noted that the high quartz content of the mixes emanates from the sand (fine aggregate) content of the ground samples.

The amorphous weight percentages represented in Table 4 for the mortar indicate the C-S-H gel formed in each mix. It can be seen how the control has an amorphous weight percentage of 33.4%, Mix 2 has 25%, Mix 3 has 29.6%, Mix 4 has 41%, and Mix 5 has 18.6%. C-S-H can significantly affect the concrete’s performance and is the main cause of the concrete’s strength [39]. Concrete’s durability is also dependent on the C-S-H gel that is formed, as it improves the microstructure of the concrete [40]. The control has a high amount of C-S-H phase due to the hydration reaction of the OPC, as C-S-H gel is the key product derived through hydration [1]. Mix 4 has the highest C-S-H phase, indicating high pozzolanic reactivity and later confirms its high-performance results (Section 3.4, Section 3.5 and Section 3.6). Mix 5 has the lowest C-S-H phase, although it has the lowest amount of portlandite phase. This could be due to 60% of the cement being replaced, causing a low content of CaO in the binder that produces a low amount of portlandite that is further consumed for pozzolanic reaction. The low amount of C-S-H phase in this mix explains why Mix 5 achieved the worst performance results (Section 3.4, Section 3.5 and Section 3.6).

#### 3.1.3. SEM

**Figure 3:**
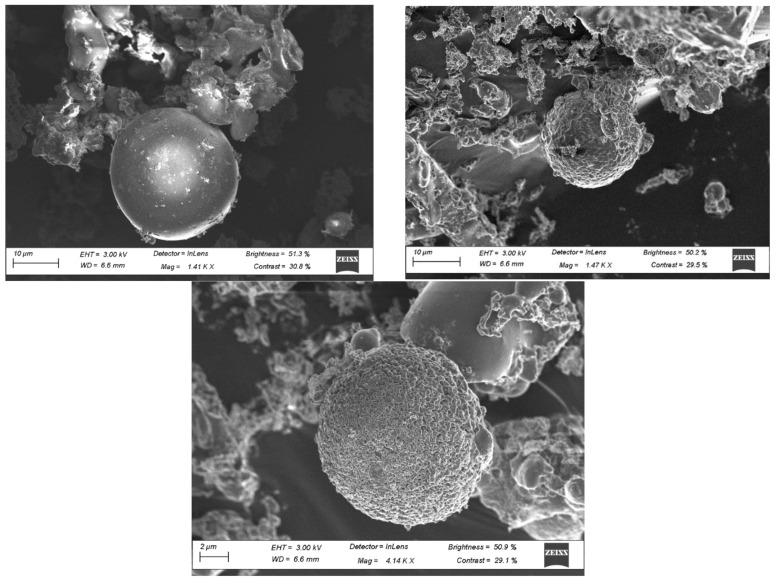
SEM results of Raw SCBA.

The SEM results obtained for the unprocessed SCBA are shown in Figure 3. The unprocessed SCBA has various particle sizes present, and most of the particles that can be seen are large and spherical, with a lot of irregularities present. The large particle sizes present in the raw SCBA are detrimental to the pozzolanic potential of the material. The study conducted by Bahurudeen et al. [5] indicated that small fine burnt particles have high pozzolanic reactivity and inert carbon-rich particles. The particles that contribute to the dark colour in Figure 3 are due to the carbon content present in the material [8]. The large presence of carbon-rich particles is due to the raw SCBA being unprocessed.

**Figure 4:**
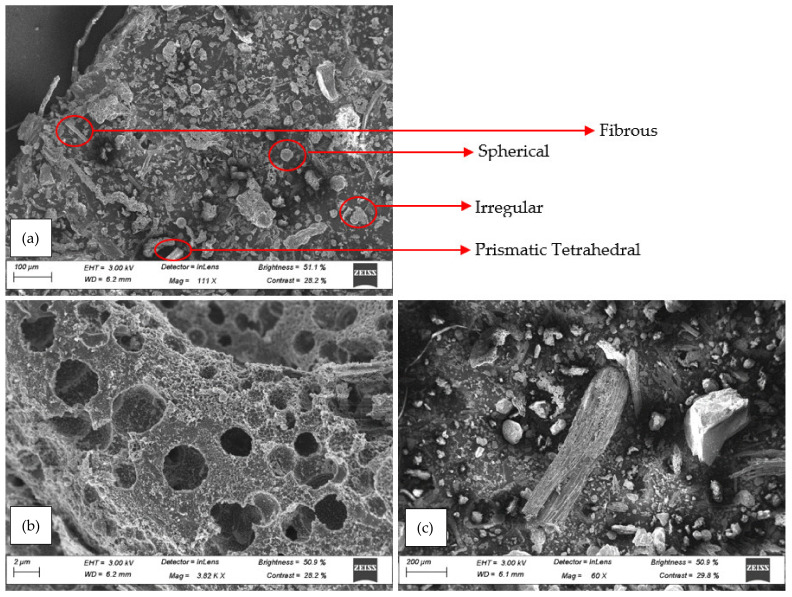
SEM images of processed SCBA: (**a**) prismatic tetrahedral spherical, fibrous, and irregular-shaped particles, (**b**) surface pores, (**c**) elongated-shaped particle with fibrous structure.

The processed SCBA SEM images (Figure 4) show that various shapes and sizes of particles can be observed, as indicated in Figure 4a. Prismatic tetrahedral shapes can be seen. These particle shapes have well-defined edges and usually correspond to the crystalline silica present in the SCBA. Other shapes that can be identified include spherical, fibrous, and irregular-shaped particles. Irregular-shaped particles have a higher surface area and are amorphous in nature, indicating a higher chance that they will react with calcium hydroxide during hydration [41]. Figure 4b shows the surface pores that are present. These pores contribute to the SCBA acting as a good water absorbent and requiring a higher water content to maintain the same consistency in blended cement concrete [8]. The blended cement concrete with a higher replacement percentage of SCBA required a larger dose of superplasticiser to maintain a similar slump. An elongated-shaped particle with a fibrous structure can be seen in Figure 4c.

These structures, normally in an elongated oval shape, have been observed to be carbon particles in SCBA [42]. The particles present in the processed SCBA are small, indicating a finer material than the raw SCBA. The literature shows finer material to be beneficial to pozzolanic performance [8,23,43].

**Figure 5:**
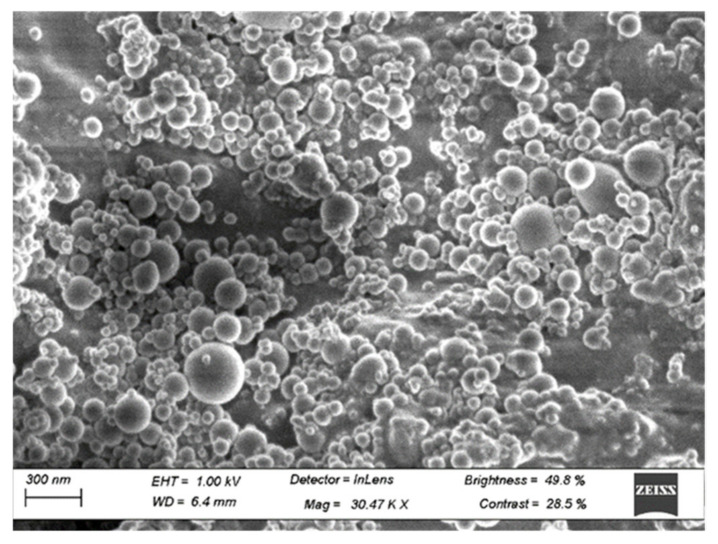
SEM results of SF.

The SF SEM results are shown in Figure 5. From these SEM images, it can be observed that most of the particles present are consistent in size and spherical in shape. Spherical-shaped particles have also been shown to be amorphous and can contribute to a higher pozzolanic potential [41]. It is also expected that the silica fume will improve the workability due to the ball-bearing effects of the particles.

### 3.2. Workability

**Figure 6:**
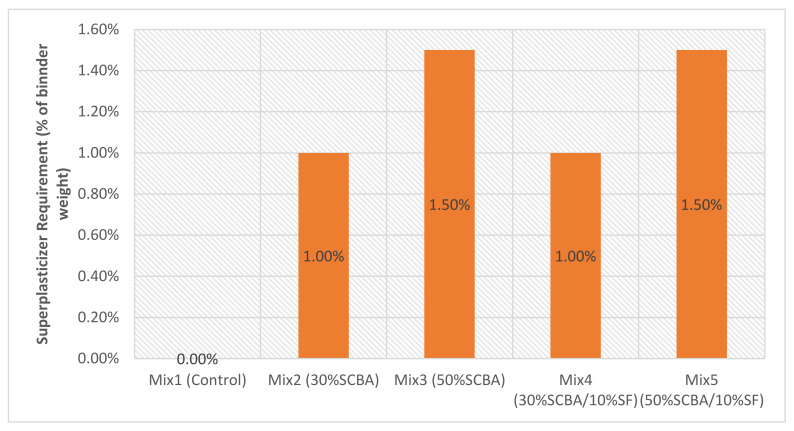
Superplasticizer dosage required per mix, as a percentage of the binder weight.

**Figure 7:**
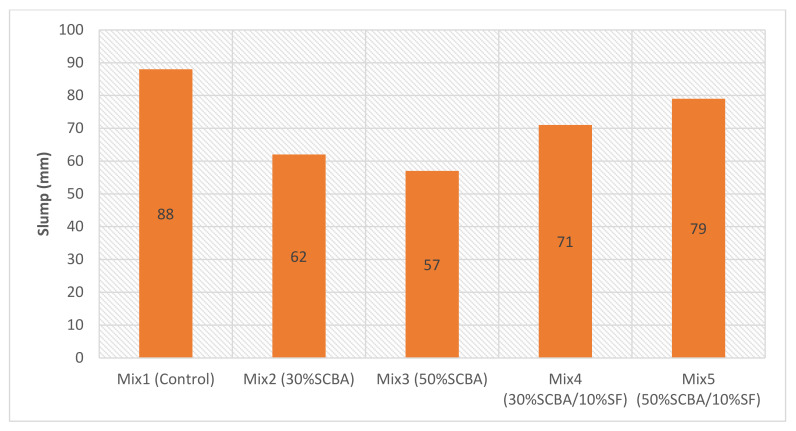
Workability obtained for each mix, in terms of slump.

The workability of every mix was designed to have a slump that falls within a range of 50–90 mm. This was achieved using different contents of the superplasticiser. Therefore, the amount of superplasticiser required varied depending on the SCM’s type and content. Figure 6 shows the dosage of superplasticiser required as a percentage of the binder weight. Figure 7 shows the workability obtained for every mix in terms of the slump. The results support the SEM results, in which mixes containing SCBA required more superplasticiser to achieve lower slump values (workability); likewise, the silica fume inclusion increases the slump values.

### 3.3. Strength Activity Index (SAI)

The results from the SAI test at 7 and 28 days of curing are 0.775 and 0.845, respectively. The results show that the 7- and 28-day SAI is above the minimum value of 0.75, stipulated in ASTM C618:2018 [34]. The SAI at 28 days indicates a 12.67 % higher value than required. This test was at 30% SCM compared to the guidelines of ASTM C311:2018 [33], stipulating 20% SCM content. Despite the higher content, the result indicates that SCBA performed well as a natural pozzolanic material.

### 3.4. Compressive Strength

**Figure 8:**
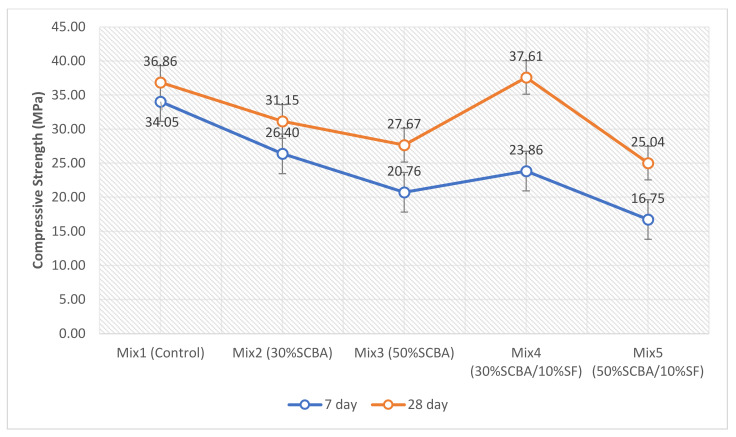
Compressive Strength results at curing ages of 7 and 28 days.

The results from the compressive strength test are shown in Figure 8. The binary blends achieved lower strength results in comparison to the control, at both curing ages. These results agree with previous studies that found that increasing the content of SCBA above 20% reduced the strength at curing ages of 7, 28, 56, and 90 days [13,15,43]. Another study observed decreasing strength above 15% content at 28 days of curing [43]. The study by Chindaprasirt et al. [44] showed that using high-volume SCBA replacement (50%) resulted in lower strength when compared to the control, at all curing ages [44]. Furthermore, a study of high-volume SCBA replacement by Rerkpiboon et al. [16] obtained similar results to Chindaprasirt et al. [44], observing a compressive strength lower than the control above 30% SCBA replacement and decreasing strength as the replacement percentage increased to 50% [16].

The main cause of the low compressive strength results obtained in high-volume SCBA blended cement concrete can be associated with low early reactivity. The high-volume SCBA causes a dilution effect, leading to delayed pozzolanic reaction, and is common to most silica-based SCMs, causing higher strength only at much later curing ages, such as 90 days [44]. The study by Rajasekar et al. [45] and Rerkpiboon et al. [16] also observed better results at all contents of SCBA, when compared to the control at a later curing age.

This study used KOH as an alkali activator to increase the early reactivity by creating a high alkaline environment. The dissolution of Al and Si from the SCBA and SF is accelerated, forming a polymeric gel that enhances early age mechanical performance [21]. The singular effect of the activator cannot be differentiated in this study because every SCM blended cement concrete mix incorporated the activator, and no comparison was made. However, pozzolanic reactivity was prevalent, which was observed by the significant increase in strength between 7 and 28 days for the SCM blended cement concretes compared to the control. Mixes 2, 3, 4, and 5 have a percentage increase of 18%, 33%, 58%, and 49% from 7 to 28 days, respectively, while the control (Mix 1) had only an 8% increase.

The improved increase between Mixes 2 and 4 and Mixes 3 and 5 also indicates that adding SF can significantly improve pozzolanic activity, rather than just SCBA. These are supported by the increased consumption of Ca(OH)_2_ from the XRD results. The SF (and alkali-activator) used allows for a good reduction (40%) of Portland cement and higher strength at 28 days. Mix 4 is adjudged to the optimum mix, with about 2% higher strength than the control. Similar results were also reported by Chindaprasirt et al. [44], which found two SCMs (fly ash and SCBA) working in synergy can produce better results than just one SCM. Similarly, the study conducted by Shannag [12] found that using SF and natural pozzolans produced better results. Mix 5 achieved a strength that is 10.5% lower than Mix 3, which is associable with the dilution effects explained in the XRD results.

### 3.5. Split Tensile Strength

**Figure 9:**
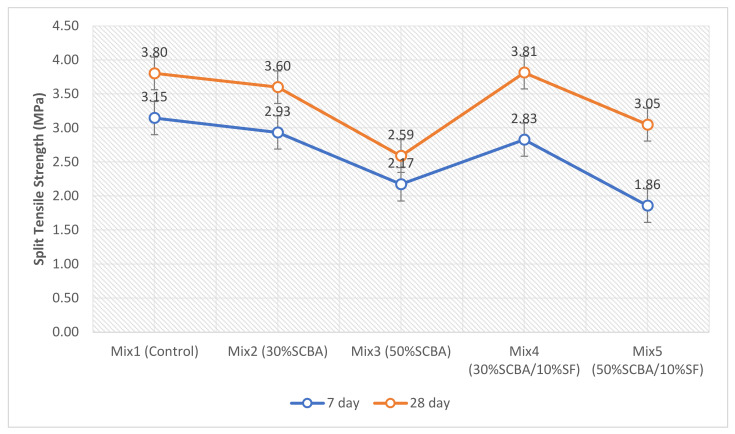
Split Tensile Strength test results at curing ages of 7 and 28 days.

The results from the split tensile strength test are shown in Figure 9. The results show a similar trend to that of the compressive strength. The binary blends achieved lower strength results in comparison to the control, at both curing ages. Previous studies on split tensile strength show similar findings for higher-volume replacements. For example, Ganesan et al. [46] found that increasing the replacement percentage of SCBA above 20% resulted in strength decreasing below the control. The study by Srinivasam and Sathiya [47] reported that split tensile strength peaked at 5%, and then above 15% replacement, strength results were lower than the control. Similarly, Batool et al. [48] reported that the split tensile strength peaked at 10% and then above this replacement, decreased below the control and continued decreasing.

The ternary blends (Mix 4 and 5) show a similar pozzolanic trend as the compressive strength. Mix 4 is observed to be the optimum mix for this test as well, achieving the highest split tensile strength out of all mixes. Mix 4 was 0.26% higher than the control at 28 days. The ternary blends achieved better results than the mix with only SCBA at 28 days. At 30% SCBA replacement, Mix 4 achieved a strength that was 5.83% higher than Mix 2. For 50% SCBA replacement, Mix 5 achieved a strength that was 17.76% higher than Mix 3. This proves the synergetic effects of SF with SCBA. The increased strength results achieved with the addition of SF in both mechanical strength tests can be attributed to the enhanced aggregate matrix bond by interfacial refinement [49] and microstructure enhancement [11]. The OPI results later support these claims.

### 3.6. Oxygen Permeability Index 

Transport properties within concrete cause different mechanisms of deterioration (mainly moisture, chloride, and oxygen) to effectively ingress through the microstructural pores of the concrete and cause durability problems. The Oxygen Permeability Index (OPI) test is a durability index that effectively assesses the resistance of concrete to oxygen permeation. The primary objective of the durability index test is to assess the cover zone that is intended to protect the reinforcement in the concrete [1]. The OPI values can be categorised by their range, presented in Table 5, to measure the durability performance expected in terms of gas permeation.

**Figure 10:**
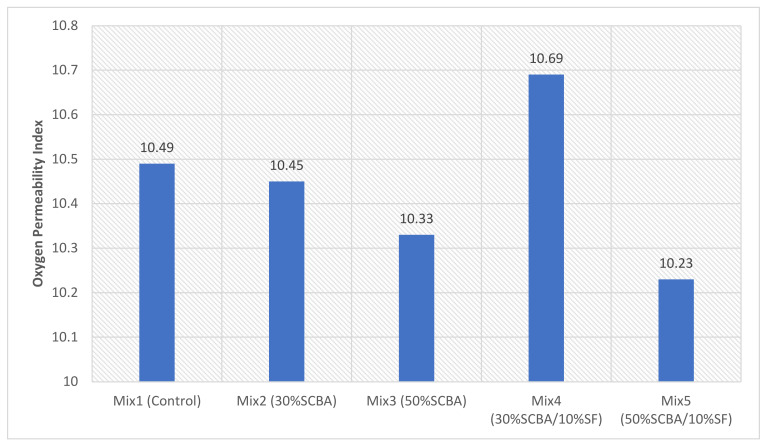
OPI results.

The results from the OPI test are shown in Table 6 and Figure 10. This test assesses the transport mechanism of gas permeation within the microstructure and macrostructure of the concrete. OPI is sensitive to the macro-porosity of the concrete, which can cause the permeating gas to find shorter paths through the concrete [50] (that is, the interconnectivity of the pores).

The results follow the same trend noticed in both the compressive and split tensile strength tests. All OPI values that have been calculated are greater than 10, which indicates that the expected durability performance in terms of gas permeation is excellent for all mixes. The binary blends (Mix 2 and 3) have lower OPI than the control and decrease as the replacement percentage increases. The ternary blend of Mix 4 achieved the highest OPI, being 1.91% higher than the control and 2.30% higher than Mix 2 (without SF). It is evident that SF helped decrease the permeability of the concrete and created more durable concrete (that is, improved interfacial refinement and microstructure enhancement). Mix 5 achieved the lowest OPI; that is, the high volume of 50% SCBA content produces concrete with a higher permeability which can be associated with less densification due to the lesser C-S-H observed in the XRD results.

### 3.7. Micro-CT Porosity

Table 7 shows the total porosity in terms of the percentage calculated for each mix, based on the total material volume and the defects’ volume. Figure 9 shows the pore size distribution by volume for each mix.

From Table 7, all SCBA blended cement concretes show a higher porosity than the control, except for Mix 2. It can, therefore, be said that above a certain limit (e.g., 30% of cement replacement), the volume content of the SCM starts to increase the porosity of the microstructure. By implication, it may become necessary to evaluate the microstructure of composites having higher volumes of SCBA. The 50% high-volume SCBA mix has the highest porosity in both the binary and ternary blends. As the SCBA content increases, the increase in porosity could be due to the irregular shape and unburnt carbon content present in SCBA [42].

**Figure 11:**
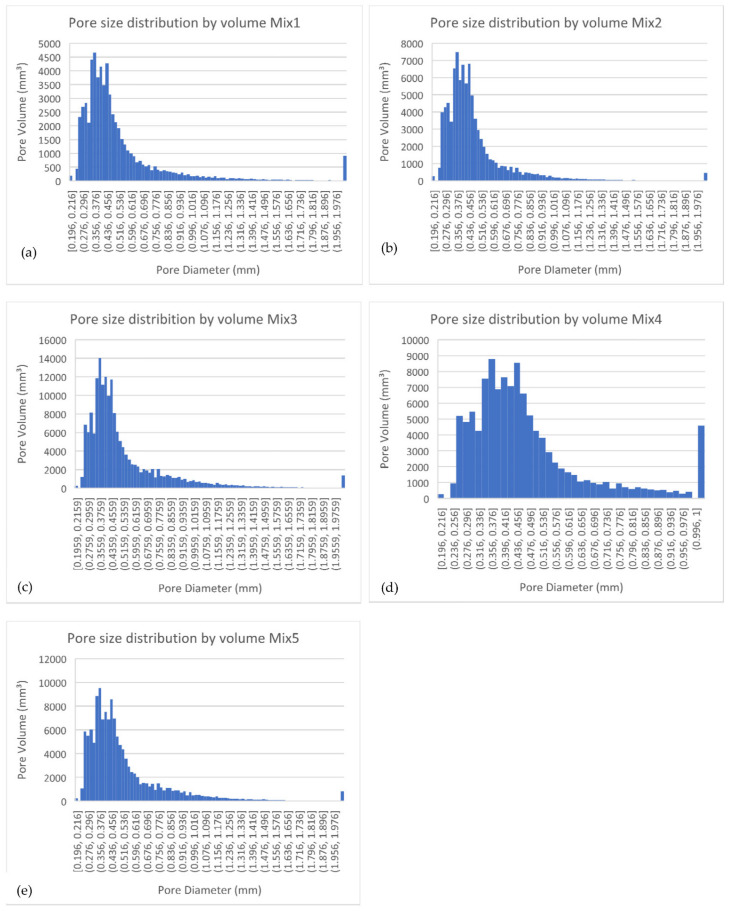
Pore size distribution by volume of (**a**) Mix 1 (**b**) Mix 2 (**c**) Mix 3 (**d**) Mix 4 and (**e**) Mix 5.

From the pore size distributions by volume in Figure 11a–e for all mixes, it is evident that the pore diameter that takes up the largest volume is in the range of 0.356–0.376 mm for every mix, indicating the critical pore size is situated in this range. For the binary blends (Mix 2 and 3), as the replacement percentage of SCBA increases, the volume of these critical pores increases. This is also the case of the ternary blends (Mix 4 and 5) as the content of SCBA increased, although not as high as the binary blends. This indicates a higher frequency of smaller pores in the higher volume SCBA blended cement concrete.

The study conducted by Naik [51] found that capillary pores and air voids can directly influence the permeability of concrete. It was determined that the permeability of concrete increases as the porosity of the concrete increases. The results from the OPI test confirm that a higher porosity of the mixes is associated with higher permeability. Mixes 3 and 5 both achieved the lowest OPI results (higher permeability to oxygen) and the highest porosity. However, Mix 4 had a higher porosity than the control but achieved the highest OPI (lowest permeability) overall. This indicates how the interconnectivity of the pores also impacts the permeability of the concrete; that is, the pores in Mix 4 have less connectivity. The SF in the mix could have improved the interfacial transition zone and caused finer pores with a higher tortuosity [44,49]. Another reason is the high amount of C-S-H phases that are present in Mix 4, causing densification and discontinuity of the pores [40].

When comparing the mechanical strength results to porosity, it is noted that Mixes 3 and 5 achieved the lowest strengths and the highest porosity. Porosity is one of the major determining factors for strength in concrete; with the decreasing compressive strength obtained with an increase in porosity [52], the interconnectivity of the pores can also create weak zones for the propagation of cracks.

Though a morphostructural investigation (SEM) on the mechanically tested mixtures were not reported, the quantitative XRD and porosity analyses corroborate the mechanical results, giving insights into the morphology and microstructure, respectively. C-S-H results from the XRD highlight the extent of cement hydration/bonding, while the oxygen permeability (mean K) and XCT scan results highlight the microstructural properties, including the interconnectivity of pores.

## 4. Conclusions and Recommendations

This study focused on evaluating the mechanical and durability performance of using a binary blend of SCBA with Portland cement in concrete and a ternary blend of SCBA, SF, and Portland cement, with the addition of an alkali activator. The conclusions that can be drawn from this study are as follows.

- Processing the SCBA through calcination resulted in a material that possessed better pozzolanic properties than the raw SCBA. The processed SCBA has a very high silica content and a low LOI. Moreover, the processed SCBA had an increase of 12.3% in the amorphous phase and a 10.8% decrease in the crystalline quartz phase.
- SF possessed exceptional pozzolanic properties, with a high silica content and a low LOI, superior to the processed SCBA. In addition, SF possesses essentially an amorphous phase at 96.1% and fine spherical particles confirmed by SEM images.
- The XRD analysis of the cement mortar samples at 21 days confirmed pozzolanic reaction due to the SCMs with decreasing portlandite content. This, with the SAI results, indicates that both SCBA and SF have good pozzolanic reactivity in concrete. The amorphous SF helped produce a higher amount of the C-S-H phase (Mix 4). This high amount of C-S-H phase helped increase the strength and improve the microstructure of the concrete, which consequently improved the durability. At 28 days of age, the compressive and tensile strength of the ternary blend supersedes that of the plain concrete, allowing for up to 40% cement reduction without detrimental effects.
- The results from the OPI test showed that all mixes achieved indices that can be considered excellent (>10). A similar trend to the the mechanical strength tests was observed in the OPI test. The SCBA binary blends resulted in a lower OPI than the control and decreased further as the replacement percentage increased. The ternary blend of Mix 4 achieved the highest OPI. This indicates that the addition of SF produces better mechanical performance and creates a more durable concrete.
- The interconnectivity of the pores has an impact on the permeability of the concrete more than the porosity. This is portrayed by Mix 4, with higher porosity than the control, although with higher resistance to oxygen permeation. Higher porosity impacts the mechanical performance of the concrete, as portrayed by Mixes 3 and 5, having the lowest strength and the highest porosity.

This study proves that when SF is added at 10% content as a ternary blend with SCBA, SCBA can be added up to 30% and still produce concrete with better mechanical and durability performance than the control (plain concrete). This research shows how the limitations of using high-volume SCBA can be curtailed (by including SF and an alkali activator). The findings presented in this study are fundamental in producing a more environmentally sustainable future of construction and can positively influence the concrete industry. Up to 40% of the cement content in the concrete can be replaced while providing enhanced performance characteristics at the same time. This will significantly reduce the clinker factor of the cement and consequently reduce carbon emissions. Further studies are required to optimise the alkali content/activation and achieve similar or higher early age strength as the control with high-volume SCBA.

## Figures and Tables

**Table 1:** Materials and characteristics.

Materials	Characteristics	
	RD	FM
Cement	3.14	-
SCBA	1.94	-
SF	2.35	-
Fine aggregate (2 mm max.)	2.62	2.62
Coarse aggregate (19 mm max.)	2.8	-
KOH	2.1	-

**Table 2:** Mix Proportions.

Mix	Cement (kg/m^3^)	SCBA (kg/m^3^)	SF (kg/m^3^)	Water (kg/m^3^)	Fine Aggregate (kg/m^3^)	Coarse Aggregate (kg/m^3^)	KOH (kg/m^3^)	Premia 310 (kg/m^3^)	w/b	Density (kg/m^3^)
Mix 1	400	-	-	200	830.68	995.56	-	-	0.5	2426
Mix 2	273.53	117.23	-	191.47	811.50	972.57	3.91	3.91	0.5	2374
Mix 3	192.42	192.42	-	188.57	799.20	957.82	3.85	5.77	0.5	2340
Mix 4	233.48	116.74	38.91	190.68	808.12	968.52	3.89	3.89	0.5	2364
Mix 5	153.30	191.63	38.33	187.80	795.92	953.89	3.83	5.75	0.5	2330

**Table 3:** Oxide composition of SCBA and SF.

	Oxide Composition (%)												
	Al_2_O_3_	CaO	Cr_2_O_3_	Fe_2_O_3_	K_2_O	MgO	MnO	Na_2_O	P_2_O_5_	SiO_2_	TiO_2_	LOI	PO
Processed SCBA	3.55	3.73	0.02	2.71	1.83	-	-	0.07	2.83	75.98	0.41	7.94	82.24
SF	1.55	1.62	-	5.10	2.53	-	-	0.39	0.17	82.00	0.03	5.77	88.65

Legend: LOI—Loss on ignition, PO—Pozzolanic Oxides (SiO_2_ + Al_2_O_3_ + Fe_2_O_3_).

**Table 4:** Relative Phase amounts (weight%).

Relative Phase Amounts (Weight%)	Mix 1 Mortar	Mix 2 Mortar	Mix 3 Mortar	Mix 4 Mortar	Mix 5 Mortar	Raw SCBA	Processed SCBA	SF
Quartz	59.1	69.9	60.3	56	79.4	57.8	47	
Calcite	1.3	0.8	0.4	0.6	0.2	0	0	
Hatrurite	1.9	0.8	0	0.2	0.4	0.1	0	
Ettringite	0.4	0.4	0.3	0.5	0.1	0	0	
Portlandite	3.4	2	0.8	0.4	0.2	0	0	
Plagioclase	0	0.9	5.2	0	0	0	0	
Chlorite	0.3	0.1	1.6	0.4	0.5	0.4	0.4	
Muscovite	0.1	0.1	1.8	0.9	0.4	0.8	1.1	
Whitlockite	0	0	0	0	0.2	4	2.2	
Quartz								0.6
Magnetite								1.2
Sylvine								1
Diopside								1
Amorphous	33.4	25	29.6	41	18.6	36.9	49.2	96.1

**Table 5:** OPI performance estimates.

	Durability Performance Estimate			
	Excellent	Good	Normal	Poor
OPI range	>10.0	9.5–10.0	9.0–9.5	<9.0

**Table 6:** Summary of OPI results.

	Mix 1 (Control)	Mix 2 (30% SCBA)	Mix 3 (50% SCBA)	Mix 4 (30% SCBA/10% SF)	Mix 5 (50% SCBA/10% SF)
Mean K (m/s)	3.492 × 10^−11^	3.582 × 10^−11^	4.838 × 10^−11^	2.287 × 10^−11^	5.868 × 10^−11^
OPI (based on average)	10.49	10.45	10.33	10.69	10.23
OPI Performance Range	Excellent	Excellent	Excellent	Excellent	Excellent

**Table 7:** Total porosity of all five mix designs.

Mix Design	Total Porosity/Defects (%)
Mix 1 (Control)	1.80
Mix 2 (30% SCBA)	1.65
Mix 3 (50% SCBA)	3.77
Mix 4 (30% SCBA/10% SF)	2.25
Mix 5 (50% SCBA/10% SF)	2.89

## Data Availability

The data provided in this study are available on request from the corresponding author.
